# Patterns and Appropriateness of Psychotropic Medications Prescribing in Primary Healthcare in Jordan

**DOI:** 10.3390/pharmacy13020044

**Published:** 2025-03-18

**Authors:** Derar H. Abdel-Qader, Alia Saleh, Abdullah Albassam, Esra’ Taybeh, Nadia Al Mazrouei, Khalid Awad Al-Kubaisi, Rana Ibrahim, Reham Aljalamdeh, Salim Hamadi, Sahar Jaradat, Shorouq Al-Omoush

**Affiliations:** 1 Faculty of Pharmacy & Medical Sciences, University of Petra, Amman 11196, Jordan; aliasalehh95@gmail.com (A.S.); shamadi@uop.edu.jo (S.H.); sahar.jar279@icloud.com (S.J.); shoroqalomoush@gmail.com (S.A.-O.); 2 Department of Pharmacy Practice, Faculty of Pharmacy, Kuwait University, Kuwait 12037, Kuwait; albassam@hsc.edu.kw; 3 Department of Applied Pharmaceutical Sciences, School of Pharmacy, Isra University, Amman 11196, Jordan; esra.taybeh@iu.edu.jo; 4 Department of Pharmacy Practice and Pharmacotherapeutics, College of Pharmacy, University of Sharjah, Sharjah 27272, United Arab Emirates; nalmazrouei@sharjah.ac.ae (N.A.M.); kalkubaissi@sharjah.ac.ae (K.A.A.-K.); ribrahim@sharjah.ac.ae (R.I.); 5 Faculty of Pharmacy, Philadelphia University, Amman 19392, Jordan; raljalamdeh@philadelphia.edu.jo

**Keywords:** psychotropic medications, community pharmacies, pharmacist interventions

## Abstract

Background: Although psychotropic medications (PMs) have enormous adverse events and may cause serious harm if administered inappropriately, there is a scarcity of research concerning the patterns and appropriateness of prescribing these medications in primary care in Jordan. This study aimed to investigate the patterns and appropriateness of PM prescription in primary care, as well as the types and frequency of pharmacist interventions in community pharmacies. Methods: A prospective observational study was conducted in 16 community pharmacies across Jordan. A data reporting sheet was developed, validated, piloted to ensure its applicability, and filled out over 12 weeks (April to June 2023), covering three regions in Jordan. Results: Overall, 426 patients with 469 prescriptions containing 919 PM orders were observed. Among the PMs prescribed, 19.4% were prescribed inappropriately. Among the PMs, 78.7% were dispensed by pharmacists. The inappropriate prescription categories (n = 178) were overprescribing (45, 25.3%), underprescribing (19, 10.7%), inappropriate medication choice (39, 21.9%), inappropriate duration of medication therapy (64, 36.0%), and inappropriate medication dosage (11, 6.2%). The top therapeutic category requested was anti-epileptics (23.9%). Conclusions: This study evaluated the prescribing patterns and appropriateness of PMs in Jordan, revealing notable instances of inappropriate PM prescriptions alongside varied and extensive pharmacist interventions.

## 1. Introduction

Psychotropic medications (PMs) are an increasingly prevalent solution to mental health disorders [1]. Major depressive disorder, anxiety disorders, bipolar disorder, and schizophrenia are just some conditions commonly treated using psychotropics as they have direct neurotransmitter-regulating properties that regulate mood disorders or manage associated symptoms [2]. 

The prescription of psychotropic medications is performed by healthcare professionals such as psychiatrists, primary care physicians, clinical pharmacists, and nurse practitioners [3]. These practitioners take account of everything including patients’ symptoms, medical history, and other situations that are relevant when they ask for PMs. Once a medication is prescribed, a community pharmacist serves as an intermediary between the prescriber and the patient, being responsible for the management of medication therapy and the safe storage conditions of medication [3].

PMs have been widely known for the effective management of these mental illness disorders; yet, some debates have arisen about their appropriate applications. The misuse, overuse, and underuse of PMs may be considered as adverse events that should always be avoided [4]. Overuse occurs when patients consume too many medications at high dosages or for extended duration [4]. Side effects, medication errors, and dependence or addicting possibilities may be the consequences of this [4]. Underuse can be described as when patients do not take medicine correctly or are provided with medicine that is inadequate or outdated. It is often the case that with improper usage, people experience adverse events and their symptoms are aggravated. However, abuse denotes those instances when someone excessively uses a drug in a way that is detrimental to themselves; this encompasses both physical dependence and psychologic dependence [4,5].

The role of the community pharmacist in mental health services is evolving quickly, with increasing levels of mental illness and the need for convenient, patient-centred care. Traditionally focused on dispensing medication, pharmacists are now being recognized more and more for their ability to influence several aspects of mental health service delivery. Experiments have shown the efficacy of training courses in enhancing pharmacists’ attitudes, knowledge, and skills in mental health, in addition to their competence in care delivery, and reducing stigma [6]. Experiments have also examined pharmacist-provided interventions such as collaborative drug therapy management and the improvement of psychotropic medication use and adherence [7]. Further, studies on prescribing antidepressants emphasize the importance of treatment duration and medication selection [8]. There are still ongoing issues, including communication with other professionals, resources, and the general problem of stigma [9,10,11]. This overview shows the mounting evidence base for incorporating community pharmacists into mental healthcare teams and stresses the need to bridge existing obstacles and optimize collaborative models.

Moreover, studies have proven that Jordanian community pharmacies offer services, such as blood pressure measurement, weight measurement, ear piercing, and first aid. Individuals rely on pharmacists for medication counselling and the management of minor ailments; hence, pharmacists are well suited to offer such advice as well as educate on different types of drugs or supplements [12].

The awareness of how PMs are dispensed by community pharmacists is crucial to identify areas in which to improve the prescribing process. By considering factors, such as patient demographics, medication type, prescriber characteristics, and pharmacy characteristics, we can recognize potential issues while devising plans to promote the appropriate use of such drugs and improve care quality while protecting the safety of mental health disorder patients. This study aimed to investigate the pattern of PM prescription in primary care in Jordan, with a focus on the appropriateness of prescription as well as the characteristics of pharmacists’ interventions in community pharmacies.

## 2. Materials and Methods

### 2.1. Study Design

A prospective observational study was conducted to collect data in a real-world setting, providing insights into the prescription practices in primary care and pharmacist interventions in community pharmacies across Jordan.

Ethical approval was granted by the Research Ethics Committee of The University of Petra to ensure adherence to standards, while safeguarding participants’ rights and well-being (O/3/8/2023).

### 2.2. Study Flow and Settings

A data reporting sheet was developed by the principal investigator after reviewing the relevant literature. The sheet was validated by three experts (psychiatrist, clinical pharmacotherapist, and pharmacist) using a scoring technique. The sheet was piloted by two pharmacists over a period of three days to check its applicability. Based on the feedback received, minor changes were made to the sheet. The reliability of the data reporting sheet was checked with the Kappa coefficient technique. This yielded a score of 0.89, indicating substantial agreement between observers. Finally, full-scale data collection was carried out by distributing the reporting sheet to pharmacists, who filled it out over a period of 12 weeks (April to June 2023).

### 2.3. Sample Size

The sample size was calculated using the formula n = (Z^2^ × P × (1 − P)) /d^2^, where Z = Z-score corresponded to the desired confidence level, P = estimated prevalence (proportion) of the outcome of interest, and d = precision [13]. Assuming a confidence level of 95% (corresponding to Z = 1.96) and a 4% rate of dispending PMs (P = 0.04) [14], in order to estimate the proportion of PMs dispensed in community pharmacies with a precision of 3%, the required sample size was 843 PMs.

### 2.4. Participants

Based on the required sample size, 16 pharmacies with 52 pharmacists were recruited to fill out the data reporting sheet over a period of 12 weeks. The selection of pharmacists was conducted to cover three regions in Jordan: Central and Capital region (Amman, Zarqa, Madaba, and Balqa), Northern region (Jerash, Irbid, Ajloun, Mafraq), and Southern region (Tafelah, Karak, Aqaba, Maan).

The inclusion criteria of pharmacies were as follows: opening at least two shifts per day and working regularly, being located near clinics, preferably those with psychiatrists, and having at least two licenced pharmacists present per shift practicing for at least one year. The pharmacies unwilling to participate were excluded. Pharmacists were given induction training on the study procedure and data collection form; this included types of medications prescribed/dispensed, and terminologies related to medication safety and pharmacist intervention classification.

### 2.5. Statistical Analysis

Data analysis was performed using SPSS version 26 [15]. In this study, the Shapiro–Wilk test was used, which showed that the collected data were normally distributed (*p* > 0.05). We reported any statistically significant differences employing the Chi-square test for categorical variables, and the independent t-test was used for continuous variables. We used regression analysis to examine how various independent variables related to a dependent variable. In this analysis, the dependent variable was categorized as either the appropriate or inappropriate prescription of PMs. A multidisciplinary committee categorized the inappropriate prescription of PMs into categories of overprescribing (the prescription of a medication for which no clear clinical indication exists), underprescribing (the omission of a potentially beneficial medication clinically indicated for the treatment or prevention of a disorder), inappropriate medication choice, inappropriate duration of medication therapy, and inappropriate medication dosage or frequency. This categorization, adopted from a previous study [16], helped us understand how different predictors influenced the likelihood of certain PM prescription practices being adopted.

## 3. Results

During the study period, 469 prescriptions containing 919 PMs were recorded. Among the prescribed PMs, 178 (19.4%) were inappropriately prescribed, and 723 were dispensed by the pharmacists (Table 1). The top three therapeutic categories prescribed were anti-epileptics (220, 23.9%), tranquillizers (105, 10.4%), and selective serotonin reuptake inhibitor (ssri) antidepressants (101, 11.0%); conversely, the least frequently prescribed categories were barbiturate plain hypnotics (14, 1.5%), non-barbiturate plain hypnotics (21, 2.3%), and nootropics (25, 2.7%).

As shown in Table 2, the highest number of PMs requested was for patients aged 52 years, with 41 PMs, followed by patients aged 25, with 40 PMs; conversely, the lowest level (2 PMs) was for patients aged 75 years.

In this study, the most common PMs prescribed were pregabalin (81, 8.8%), carbamazepine (74, 8.1%), Levetiracetam (69, 7.5%), and Clonazepam (67, 7.3%). On the other hand, the least common PMs prescribed were cyanocobalamin (6, 0.7%) and venlafaxine (7, 0.8%) (Table 3).

In our study, 178 PMs were inappropriately prescribed. The inappropriate prescription categories were overprescribing (45, 25.3%), underprescribing (19, 10.7%), inappropriate medication choice (39, 21.9%), an inappropriate duration of medication therapy (64, 36.0%), and inappropriate medication dosage (11, 6.2%) (Table 4).

Of the 2799 pharmacist interventions conducted during the study, 1023 (36.5%) were related to patient education, 362 (12.9%) were about asking for more clarification from the patient, and 214 (7.6%) involved changing the brand name of the medication prescribed (Table 5).

Among the patient education recommendations, 313 (30.6%) highlighted potential side effects, 163 (15.9%) explained the importance of medication adherence, and 122 (11.9%) were about advising the patient on lifestyle modifications that can support their mental health (Table 6).

The logistic regression model showed that physicians in the Central and Capital region were less likely to prescribe PMs inappropriately compared with those in the Northern region (AOR: 0.71; 95%CI: 0.01–0.87, *p* = 0.001) (Table 7). In addition, patients aged below 55 were more likely to be inappropriately prescribed PMs compared with those above 55 years (AOR: 1.75; 95%CI: 1.13–4.56, *p* = 0.018). Furthermore, the findings showed that SNRI antidepressants were less likely to be inappropriately prescribed compared with anti-epileptics (AOR: 0.62; 95%CI: 0.08–0.91, *p* = 0.014).

## 4. Discussion

While community pharmacists play an integral role in ensuring the safe use of PMs, research focusing on them remains lacking. This knowledge gap illustrates why urgent steps must be taken to investigate and develop evidence-based practices that empower community pharmacists to enhance patient outcomes within community settings. Therefore, this study aimed to investigate the pattern of PM prescription in primary care in Jordan, focusing on the appropriateness of prescription as well as the characteristics of pharmacists’ interventions in community pharmacies.

While limited to the pharmacies sampled, the frequent prescription of PMs observed in this study may reflect the high prevalence of treated mental disorders within the specific regions of Jordan included in our analysis. While there has been no study addressing this problem in the community sector of Jordan, there was a small study that reviewed each medical record of 67 patients with psychiatric disorders that found a high rate of prescriptions for PMs [17]

Our results coincided with previous research suggesting that antiepileptics, tranquillizers, and selective serotonin reuptake inhibitor (SSRI) antidepressants were the most common drugs prescribed to combat anxiety and major depressive disorders. These results backed what was known and proved the effectiveness of PMs for managing mental disorders. PMs were mostly prescribed to those aged between 52 years and 25 years, which indicated that they might be more vulnerable to mental health disorders. This finding was confirmed by the existing literature that emphasized the vulnerability to particular types of disorders during life stages. The population aged between 18 and 29 years have been identified with the highest prevalence rate of anxiety disorders, while those from 45 to 64 years old are more susceptible to depression [18]. Another research from Latin America [19] found that the highest number of PMs was prescribed for people at the age of 58. To develop age-tailored treatment plans and interventions, it is necessary to consider the variations that may only be applicable to these groups.

This alarming finding of a high inappropriate prescription incidence in our study (19.4%) was an issue that warranted prompt targeted action. This evidence almost paralleled the results from other countries that established an upsurge of PM prescription for psychiatric disorders management [20]. In a Norwegian study [20], the inappropriate prescription of PMs, based on the prescribing quality indicators, was compared in three Norwegian nursing homes in 2000 and 2016 and it was found that the potential inappropriate prescription of PMs increased from 0.8% to 27.9%.

Regarding the possible causes of inappropriate prescription by healthcare professionals, one possible explanation is that some professionals might have a wrong or outdated understanding of mental health disorders and pharmacological management guidelines. The other explanation could be that there were many patients with no access to specialized mental healthcare clinics, where they had to refer to non-psychiatrists.

Our findings showed that providers’ location, patients’ age, and the type of PMs predicted the occurrence of inappropriate prescription. In detail, inappropriate prescription was lower in the Central and Capital region than in the Northern region, possibly reflecting the variation among physicians in Jordan with respect to the availability of mental health teaching resources, physicians’ level of training, and cultural attitudes towards mental illnesses.

Furthermore, younger patients were more likely to be prescribed PMs inappropriately than those older than 55 years. This may reveal that younger people may administer PMs more frequently and incorrectly for a variety of reasons, including family pressure on physicians to prescribe PMs to control their children’s challenging behaviours.

Furthermore, the fact that SNRI antidepressants were less likely to be prescribed inappropriately than anti-epileptics may indicate that certain PMs might be more prone to misuse by physicians than others based on factors such as the safety profile of the medication, the availability of alternative treatment options, or clinical guidelines for treating mental health conditions.

These factors aligned somewhat with previous studies from other countries. One study conducted in the US found that older age, “seen before” status, and antidepressant drug class were factors making one less likely to have PMs prescribed inappropriately, while enrollment in Medicaid, antipsychotic drug class, living in the Northeast region, and receiving healthcare in a metropolitan area were negatively associated with PM prescription [21].

The findings of our study indicated that pharmacists performed a high number of interventions on prescriptions with PMs, which underscored the evolving role of community pharmacists in mental health. Additionally, there were three major types of interventions, namely patient education, asking for more clarifications from patients, and changing the brand name of prescribed medications.

Pharmacists’ roles in mental health have been shown to have positive effects, leading to improved prescribing practices and satisfaction among those living with mental illness [22]). Nonetheless, studies are often of low quality, necessitating well-designed randomized controlled trials (RCTs) that not only demonstrate effectiveness but also assess cost-effectiveness in different healthcare settings, including the community, hospitals, and others [22,23].

Studies have demonstrated the efficacy of pharmacist-led interventions in improving antidepressant medication adherence through education and monitoring [24,25]. Some studies investigated screening mental illnesses by pharmacists; investigations included perinatal depression screening as well as pilot studies, showing their feasibility [26,27].

A recent systematic review on pharmacist-led depression screening practices among adults found that they could use screening tools to detect undiagnosed depression in adult populations; however, more robust and high-quality research in this area should be conducted in order to ascertain cost-effectiveness and the clinical implications of such screening practices [28].

## 5. Conclusions

This study investigated the prescription patterns and appropriateness of PMs in primary care in Jordan. To sum up, our study revealed high rates of the prescription of potentially inappropriate PMs, indicating a concerning trend. Patient age, the location of the clinic, and drug class were associated with inappropriate prescription. Pharmacist interventions were frequently documented.

The strengths of this study came from its uniqueness, as there have been no studies investigating the frequency, types, and appropriateness of PMs in primary care in the region. One more strength was the observational nature of this study, which added to the validity of the findings. However, the study had some limitations. The data collection relied on pharmacist reporting and was potentially subject to recall bias or selective reporting, where pharmacists may have been more inclined to document interventions or problematic prescriptions. Prescriptions without interventions or issues may have been under-reported, potentially overestimating the rate of inappropriate prescription. While training was provided to pharmacists on using the data collection form, variations in interpretation and documentation practices cannot be ruled out.

## Figures and Tables

**Table 1:** Categories of PMs.

Variable	Total, n (%)
Number of prescriptions containing PMs, n	469
Number of PM orders in prescriptions, n	919
PMs dispensed by the pharmacist, n	723
Drug category of PMs requested *	
Anti-epileptics	220 (23.9%)
Tranquillizers	105 (11.4%)
SSRI antidepressant	101 (11.0%)
Atypical antipsychotics	96 (10.4%)
Antidepressants (other)	51 (5.5%)
SNRI antidepressants	61 (6.6%)
Anti-Parkinson preparations	45 (4.9%)
Psycholeptics	39 (4.2%)
Non-barbiturate plain hypnotics and sedatives	21 (2.3%)
Conventional antipsychotics	75 (8.2%)
Nootropics (drugs that enhance cognitive function)	25 (2.7%)
Psychostimulants	34 (3.7%)
Barbiturate plain hypnotics and sedatives	14 (1.5%)
Mood stabilizers	32 (3.5%)

PM: psychotropic medication. Drug classes determined according to Anatomical Therapeutic Chemical (ATC) codes. SSRI: selective serotonin reuptake inhibitor. SNRI: serotonin–norepinephrine reuptake inhibitor.

**Table 2:** Number of PMs requested per age of patients (N = 919).

Age (Year)	Number of Psychotropic Medications Requested
15	9
16	6
17	17
18	11
19	16
20	19
21	18
22	11
23	28
24	31
25	40
26	26
27	20
28	33
29	16
30	34
31	11
32	16
33	9
34	13
35	11
36	22
37	11
38	14
39	17
40	11
41	12
42	8
43	6
44	8
45	9
46	38
47	11
48	19
49	32
50	21
51	17
52	41
53	11
54	9
55	6
56	18
57	22
58	16
59	22
60	14
61	6
62	11
63	13
64	8
65	7
66	3
67	12
68	11
69	5
70	3
71	6
72	8
73	7
74	7
75	2

**Table 3:** Appropriateness of PMs prescribed (N = 919).

Drug	Total	Appropriately Prescribed	Inappropriately Prescribed
Pregabalin	81	74	7
Carbamazepine	74	68	6
Levetiracetam	69	52	17
Clonazepam	67	44	23
Gabapentin	65	57	8
Valproic acid	58	50	8
Escitalopram	56	50	6
Zopiclone	41	35	6
Citalopram	33	18	15
Olanzapine	33	28	5
Alprazolam	30	28	2
Sertraline	29	24	5
Lorazepam	29	16	13
Triazolam	28	20	8
Fluoxetine	28	26	2
Diazepam	25	21	4
Paroxetine	23	14	9
Levodopa/Carbidopa	18	17	1
Oxcarbazepine	17	11	6
Phenobarbital	16	14	2
Fluvoxamine	14	11	3
Quetiapine	12	9	3
Risperidone	12	8	4
Lithium carbonate	11	8	3
Haloperidol	10	6	4
Zolpidem	8	7	1
Venlafaxine	7	6	1
Cyanocobalamin	6	4	2
Other	19	15	4

**Table 4:** Categories of inappropriate prescription of PMs (N = 178).

Categories of Inappropriate Prescription of PMs	Total, n (%)
Inappropriate duration of medication therapy	64 (36.0%)
Overprescribing	45 (25.3%)
Inappropriate medication choice	39 (21.9%)
Underprescribing	19 (10.7%)
Inappropriate medication dosage	11 (6.2%)

**Table 5:** Pharmacist interventions (N = 2799).

Parameter	Total, n (%)
Did not dispense *	196 (7.0%)
Dispense **	723 (25.8%)
Ask for clarifications from the patient	362 (12.9%)
Ask for clarifications from the physician	186 (6.6%)
Increasing the dose	24 (0.9%)
Lowering the dose	71 (2.5%)
Changing the brand name of the medication prescribed	214 (7.6%)
Patient education	1023 (36.5%)

* PMs with serious errors, which were referred back to the physician. ** PMs with no errors, where patients did not ask or need any counselling.

**Table 6:** Types of patient education (N = 2799).

Parameter	Total, n (%)
Highlighting potential side effects	313 (30.6%)
Explaining the importance of medication adherence	163 (15.9%)
Discussing the dosage instructions, frequency of administration, and duration of treatment.	111 (10.9%)
Instructing the patient on the correct way to take the medication	108 (10.6%)
Discussing any special storage requirements	65 (6.4%)
Providing strategies to manage common side effects ***	72 (7.0%)
Advising the patient to report any severe or persistent side effects	69 (6.7%)
Advising the patient on lifestyle modifications that can support their mental health	122 (11.9%)

*** common side effects, such as nausea, drowsiness, or dry mouth.

**Table 7:** Predictors of inappropriate PM prescribing.

Predictors (Independent Variables)	AOR	95% CI		*p* Value
		**Lower**	**Upper**	
**Location**				
Central and Capital region vs. Northern region	0.71	0.01	0.87	**0.001**
Southern region vs. Northern region	0.86	0.12	1.02	0.096
**Patient age**				
Below or equal 55 vs. above 55	1.75	1.13	4.56	**0.018**
**Drug class**				
SSRI antidepressant vs. anti-epileptics	0.92	0.56	1.79	0.308
Tranquillizers vs. anti-epileptics	1.13	0.84	2.66	0.228
Atypical antipsychotics vs. anti-epileptics	0.58	0.01	4.65	0.338
Antidepressants (other) vs. anti-epileptics	0.86	0.28	3.55	0.177
SNRI antidepressants vs. anti-epileptics	0.62	0.08	0.91	**0.014**
Anti-Parkinson preparations vs. anti-epileptics	1.96	0.128	5.66	0.344
Psycholeptics vs. anti-epileptics	2.86	0.56	7.88	0.506
Non-barbiturate plain hypnotics and sedatives vs. anti-epileptics	0.98	0.25	8.61	0.691
Conventional antipsychotics vs. anti-epileptics	1.56	0.68	3.16	0.181
Nootropics (drugs that enhance cognitive function) vs. anti-epileptics	2.58	0.91	17.66	0.089
Psychostimulants vs. anti-epileptics	0.63	0.17	1.09	0.091
Barbiturate plain hypnotics and sedatives vs. anti-epileptics	3.56	0.08	22.31	0.118
Mood stabilizers vs. anti-epileptics	3.87	0.57	16.62	0.322

AOR: Adjusted odds ratio. Bold *p*-values indicate significant results.

## Data Availability

Data is unavailable due to privacy or ethical restrictions.
